# Molecular Reclassification of Crohn's Disease by Cluster Analysis of Genetic Variants

**DOI:** 10.1371/journal.pone.0012952

**Published:** 2010-09-23

**Authors:** Isabelle Cleynen, Jestinah M. Mahachie John, Liesbet Henckaerts, Wouter Van Moerkercke, Paul Rutgeerts, Kristel Van Steen, Severine Vermeire

**Affiliations:** 1 Department of Gastroenterology, KU Leuven, Leuven, Belgium; 2 Systems and Modeling Unit, Department of Electrical Engineering and Computer Science, University of Liège, Liège, Belgium; 3 Bioinformatics and Modeling, GIGA-R, University of Liège, Liège, Belgium; 4 Department of Medicine, UZ Leuven, Leuven, Belgium; HelmholtzZentrum München, Germany

## Abstract

**Background:**

Crohn's Disease (CD) has a heterogeneous presentation, and is typically classified according to extent and location of disease. The genetic susceptibility to CD is well known and genome-wide association scans (GWAS) and meta-analysis thereof have identified over 30 susceptibility loci. Except for the association between ileal CD and *NOD2* mutations, efforts in trying to link CD genetics to clinical subphenotypes have not been very successful. We hypothesized that the large number of confirmed genetic variants enables (better) classification of CD patients.

**Methodology/Principal Findings:**

To look for genetic-based subgroups, genotyping results of 46 SNPs identified from CD GWAS were analyzed by Latent Class Analysis (LCA) in CD patients and in healthy controls. Six genetic-based subgroups were identified in CD patients, which were significantly different from the five subgroups found in healthy controls. The identified CD-specific clusters are therefore likely to contribute to disease behavior. We then looked at whether we could relate the genetic-based subgroups to the currently used clinical parameters. Although modest differences in prevalence of disease location and behavior could be observed among the CD clusters, Random Forest analysis showed that patients could not be allocated to one of the 6 genetic-based subgroups based on the typically used clinical parameters alone. This points to a poor relationship between the genetic-based subgroups and the used clinical subphenotypes.

**Conclusions/Significance:**

This approach serves as a first step to reclassify Crohn's disease. The used technique can be applied to other common complex diseases as well, and will help to complete patient characterization, in order to evolve towards personalized medicine.

## Introduction

Crohn's disease (CD) is a heterogeneous disorder with differences in severity, location, behavior and age at onset of inflammation. The heterogeneity of the disease has important implications towards clinical management: patients with a more severe disease course might benefit from early introduction of immunomodulators and/or biologicals, while patients with favorable disease prognosis could be spared from intense treatment and possible side-effects.

The genetic background of CD has been extensively evaluated. This has led to significant insights into the mechanism of the disease, such as a disturbed surveillance of bacteria of the microflora by the intestinal mucosa (*NOD2*), dysregulation of adaptive immunity (*IL23R*), or deficient autophagy (*ATG16L1*, *IRGM*). Meta-analysis of three CD GWAS have identified more than 30 loci associated to CD, with odds ratios (OR) ranging from 1.08 to 3.99 [1]. As a general principle of complex traits, particular disease associated variants are found with increased frequency in patients when compared to controls. However, these variants appear neither unique nor necessary for the disease to express itself. Furthermore, attempts have been made to link the associated genetic variants with the classic clinical CD subphenotypes. A clear association has been found for *NOD2/CARD15* variants and ileal disease location [2]–[5]. However, for none of the other susceptibility genes, a robust association with any of these clinical subphenotypes could be shown. Our group recently examined whether the associated genes or a combination thereof could predict clinical outcome of CD. We showed that presence of risk alleles at some of the CD-associated genetic loci influenced disease progression, but overall the predictive power of these risk alleles was fairly poor [6].

The genetic contribution to Crohn's disease is without a doubt, as is the fact that not all CD patients have the same disease course. Although there are little robust genotype-phenotype associations described for CD, we hypothesize that subgroups of CD patients do exist on a molecular level. Here we report on our efforts to reclassify Crohn's disease into subgroups based on currently confirmed genetic markers. We then analyzed whether these subgroups are associated with the classically used clinical subphenotypes.

## Methods

### Study samples

875 CD patients previously described in [6] were included in this study. In brief, patients were recruited in the framework of the inflammatory Bowel Disease (IBD) genetics study that started in 1997 at the IBD unit of the University Hospital in Leuven (Belgium). Patients were unrelated and of Western European origin. Diagnosis of CD was based on the most recent international classification (Montreal classification) [7], [8]. Median time of follow-up since diagnosis is 14 years (IQR 7–22 years). The control group consisted of 367 unrelated healthy volunteers (healthy control HC) of Western European origin, without a family history of IBD or other immune related disorders. Ethical approval was given by the Ethics Board of the University Hospital Leuven, and written informed consent was obtained from all participants. Samples and data were stored in a coded, anonymized database. Patient files were reviewed for phenotypic information as described in [6] (also see Table 1, ‘Overall’).

**Table 1:** Characteristics of all included CD patients (Overall), and of the six genetic-based subgroups (Cluster A–F).

Genetic-based subgroup	Overall	Cluster A	Cluster B	Cluster C	Cluster D	Cluster E	Cluster F
*Subjects n*	875	302	96	62	117	59	239
*Gender n(%)*							
Male	360 (41%)	120 (40%)	44 (46%)	30 (48%)	52 (44%)	25 (42%)	89 (37%)
Female	515 (59%)	182 (60%)	52 (54%)	32 (52%)	62 (56%)	34 (58%)	150 (63%)
*Median age at diagnosis (Q25–Q75)*	24 (18–31)	23 (18–31)	25 (18–32)	25 (19–32)	23 (17–30)	24 (20–32)	25 (18–33)
*Location n(%)*							
Colon§	113 (13%)	34 (11%)	15 (16%)	3 (5%)	17 (15%)	9 (15%)	35 (15%)
Ileum§	326 (37%)	107 (35%)	36 (38%)	32 (52%)	46 (39%)	19 (32%)	86 (36%)
Ileocolonic§	433 (50%)	161 (53%)	44 (46%)	27 (44%)	54 (46%)	31 (53%)	116 (49%)
Anal	331 (38%)	118 (39%)	33 (34%)	26 (42%)	51 (44%)	15 (25%)	88 (37%)
*Behavior n(%)*							
Inflammatory*	430 (49%)	144 (48%)	57 (59%)	23 (37%)	60 (51%)	30 (51%)	116 (49%)
Stenosing*	329 (38%)	115 (38%)	33 (34%)	31 (50%)	36 (31%)	18 (31%)	96 (40%)
Non-perianal fistulae	227 (26%)	82 (27%)	16 (17%)	18 (29%)	33 (28%)	16 (27%)	62 (26%)
Perianal fistulae*	267 (31%)	73 (29%)	31 (32%)	26 (42%)	34 (29%)	17 (29%)	73 (31%)
*Surgery* # * n(%)*	493 (57%)	171 (57%)	54 (56%)	45 (73%)	62 (53%)	31 (53%)	130 (54%)

§ three missing values. Colonic, ileal, and ileocolonic location add up to 100% (are mutually exclusive). Anal disease can occur together with any of the other locations, and thus represents the % of patients in the respective cluster that also has anal involvement.

* one missing.

# four missing.

### DNA extraction and genotyping

DNA extraction and genotyping was performed as described earlier [6]. A total of 46 markers identified from different GWAS performed on CD, and/or meta-analysis of these GWAS [1], were included in this study. Table S1 includes a list of all SNPs, with the reference where the SNP was selected from, as well as the odds ratio found in that study. In Table S2, allele and genotype counts in the CD patients and HCs studied here are listed. The minor allele was defined as the less frequent allele in the control group. For the analysis performed in this study, coding for the additive genetic model was used with wild-type individuals (homozygous for the major allele) coded as 0, heterozygous individuals as 1, and individuals homozygous for the minor allele as 2.

### Statistical analyses

Detailed information on the used statistical analyses can be found in the supporting information (Methods S1) online.

#### Cluster identification

To identify clusters, latent class analysis (LCA) was applied to the set of 46 genetic markers genotyped in CD patients and healthy controls. Analyses were performed in the patient and control group separately. LCA assumes that the population is composed of sub-populations (latent classes), each having its distinctive distribution of the included variables [9].

LCA was performed with Multimix, which can handle both continuous and categorical variables [10], as well as missing data [11]. Hence, for this study all individuals - including those with missing genotypes - were included. Class assignment was based on posterior probabilities. In particular, individuals were allocated to classes or clusters on the basis of highest membership probability. The number of latent classes (N) was derived from bootstrap p-values for the likelihood ratio (LR) test with the null hypothesis that the population is ‘best’ explained by N classes. The alpha level used for determining the number of latent classes is 0.05. Per model, 20 bootstrap samples were generated, making sure that the same percentages of missingness as in the original sample were attained.

#### Construction of classification trees

Once subjects were grouped into classes or clusters, on the basis of their available genetic information, we determined the genetic markers that contributed most to the formation of the clusters. To perform this step, i.e. to gain insight into the meaning of the formed clusters, classification trees were generated with the R rpart package (R 2.9.1). Hence, trees were grown using the cluster variable obtained from Multimix as the (categorical) response and SNPs as potential explanatory variates. Goodness-of-fit of the obtained classification tree was compared to the cluster assignment of Multimix, by dropping individuals down the classification tree, and by comparing the R tree-based classification of subjects to the Multimix-based one.

#### Testing of the hypothesis of no overall status effect (case vs. control)

To check for the effect of status (case vs. control), canonical discriminant analysis (CDA) was applied to LCA data of both CD patients and healthy controls (SAS 9.1.3). Two analyses were performed: (analysis 1) To globally look at the differences between CD patients and healthy controls, canonical variables were calculated for CD patients only (say Y1, Y2), and for healthy controls only (say X1, X2). In CDA, Y1,Y2 and X1,X2 are chosen so as to maximize separation between the clusters. (analysis 2) To test whether there is a difference in terms of clustering spread/separation rule between CD patients and healthy controls, canonical variables were computed for CD patients only (say Y1, Y2). The values of Y1 and Y2 were subsequently computed for the healthy controls using the “linear combination” rule derived for the CD patients. Mean values of Y1 and Y2 between CD patients and healthy controls were then compared using MANOVA.

#### Association of genetic-based subgroups and clinical characteristics

Univariate Chi-squares (or Fisher Exact tests when necessary) were performed using SPSS 15.0 to test for associations between each of the cluster memberships and the clinical subphenotypes gender; colonic, ileal, ileocolonic disease location at last follow-up; anal disease at last follow-up; inflammatory, stricturing, non-perianal fistulizing, or perianal fistulizing disease behavior at last follow-up; and need for surgery. Multiple logistic regression (using automated backward variable selection) was performed including those clinical parameters that showed at least a trend towards significance in univariate analysis (cut-off p-value = 0.1) using SPSS 15.0. These tests were complemented with Random Forest (RF) analysis in R (2.9.1; http://www.stat.berkeley.edu/~breiman/RandomForests/cc_home.htm). RF analysis estimates the importance of variables in determining classification and is able to highlight possible variable interactions.

## Results

### Cluster identification in CD patients

Latent class analysis (LCA) identified 6 genetic-based subgroups (clusters) in CD. For each patient, membership probabilities for each cluster were computed. As mentioned above, an individual was assigned to the cluster for which they had the highest probability. 90% of CD patients (n = 788) had a highest membership probability of >0.9, indicating clear membership. For 4% of CD patients (n = 37) the highest membership probability was <0.6, indicative of more uncertain membership (see Table S3 for all membership probabilities).

**Figure 1:**
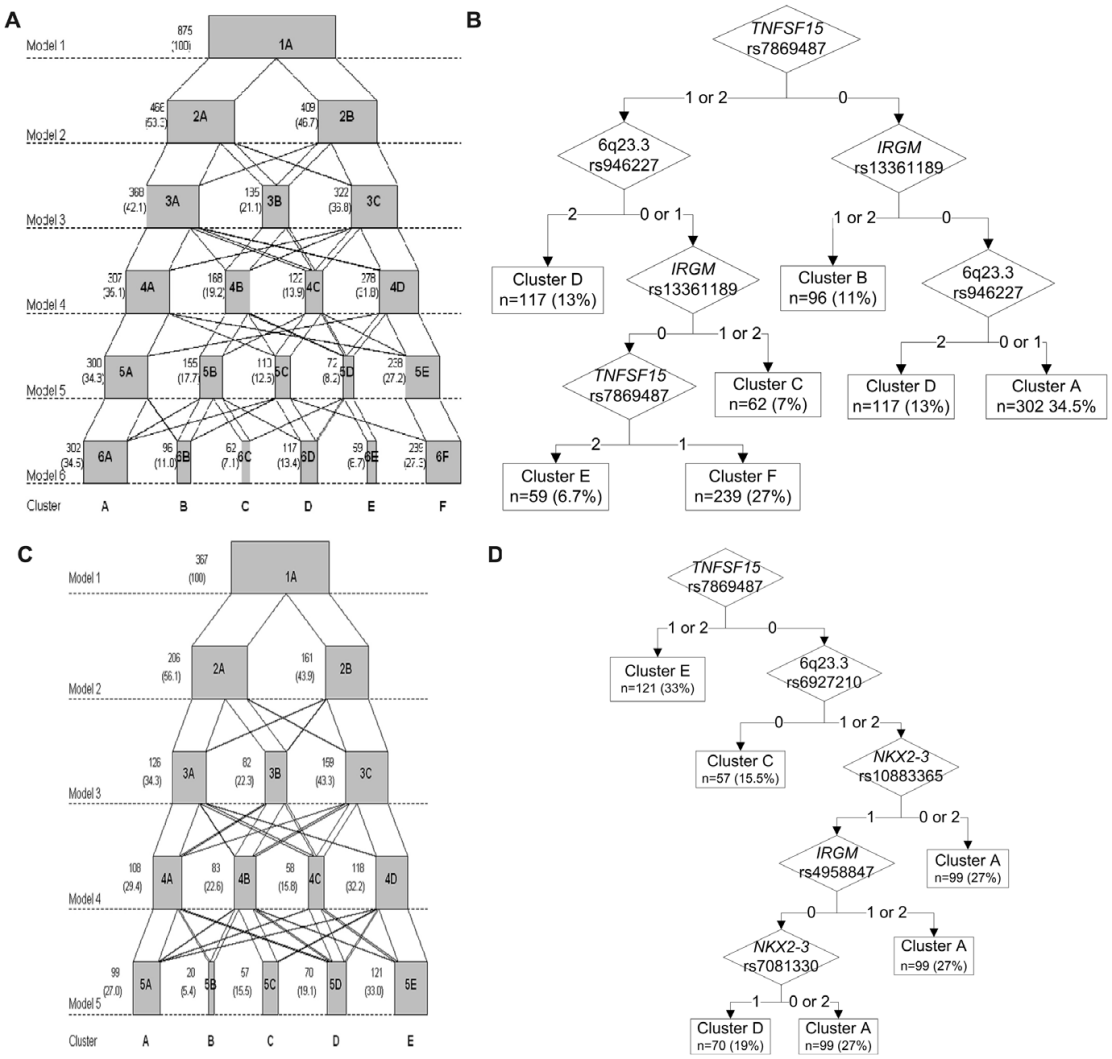
Modelbuilding process and classification trees for the final model. Formation of genetic-based subgroups when the number of clusters was increased stepwise for CD patients (**panel A**) or healthy controls (**panel C**) is shown. Data are presented as n (%). Box widths are proportional to the number of individuals in the respective cluster. The area of the white parallelograms connecting two clusters, one from Model i and one from Model i+1, is proportional to the number of individuals that are common between these clusters. Tree plot showing how SNPs determine the grouping of individuals into the different clusters for CD patients (**panel B**) and healthy controls (**panel D**). The diamond indicates where a decision is made (genotype 0 (wild-type), 1 (heterozygous) and/or 2 (homozygous mutant)). A rectangle indicates the decision (which cluster the patient belongs to when following the tree). The number and percentage of individuals in each cluster is presented as well.

To investigate the relationship between subgroups identified in the different steps of the model building, the number of CD patients “flowing” from a cluster identified in one step (‘Model i’ (i≥1), e.g. a model with one cluster) to a cluster identified in the next step (‘Model i+1’, e.g. a model with two clusters) was determined (Figure 1A). Notably, the majority of patients who belong to a particular cluster in ‘Model i’, tend to be redistributed to a single cluster in ‘Model i+1’, as can be observed by inspecting connected clusters in Figure 1A: the white parallelograms connecting two clusters, one from ‘Model i’ and one from ‘Model i+1’, the area of which is proportional to the number of individuals that are common to the clusters.

The grouping into the 6 CD clusters was *best* explained by 3 SNPs (rpart analysis; Figure 1B): rs7869487 in *TNFSF15*, rs13361189 in *IRGM*, and rs946227 located on 6q23.3.

When dropping the CD patients down the SNP tree, only 39 CD patients (4.5%) were misclassified compared to the multimix cluster allocation. A total of 58 CD patients (6.6%) could not be allocated to any of the CD subgroups because of missing genotypes for at least one of the determining SNPs.

### Cluster identification in control samples

If patient genotypes would cluster purely at random, then the same clusters are to be expected in the control samples. We therefore ran the Multimix program (LCA) also on 367 healthy controls (HC). A model with 5 clusters best explained the heterogeneity in healthy controls. A total of 83% of HC (n = 305) had a highest membership probability of >0.9, indicating clear membership. For 4.6% of HC (n = 17) the highest membership probability was <0.6, which is indicative of highly uncertain cluster membership assignments (data not shown).

The “flow” of control individuals through the clusters and formation of the clusters in the stepwise models is shown in Figure 1C. As we observed for the CD patients LCA analysis, identified clusters appeared to be stable across the different models.

Tree building with rpart showed that the following SNP combinations *best* explained the control clusters (Figure 1D): rs7869487 in *TNFSF15*, rs6927210 on chromosome 6, rs10883365 and rs7081330 in *NKX2-3*, and rs4958847 in *IRGM*. Cluster B could not be explained by specific SNPs, and may be interpreted as a waste bin: with the available genetic markers, no SNP appeared to be more important than others to explain cluster B.

We observed that 79 healthy controls (21.5%) were misclassified. Moreover, 39 HC (10.6%) were not allocated to a control cluster because of missing genotypes in at least one of the *best* determining SNPs, when dropped down the control SNP tree. This high prediction error rate is partly explained by the lack of any SNP-based rule for cluster B.

### Comparing formed clusters between CD patients and healthy controls

**Figure 2:**
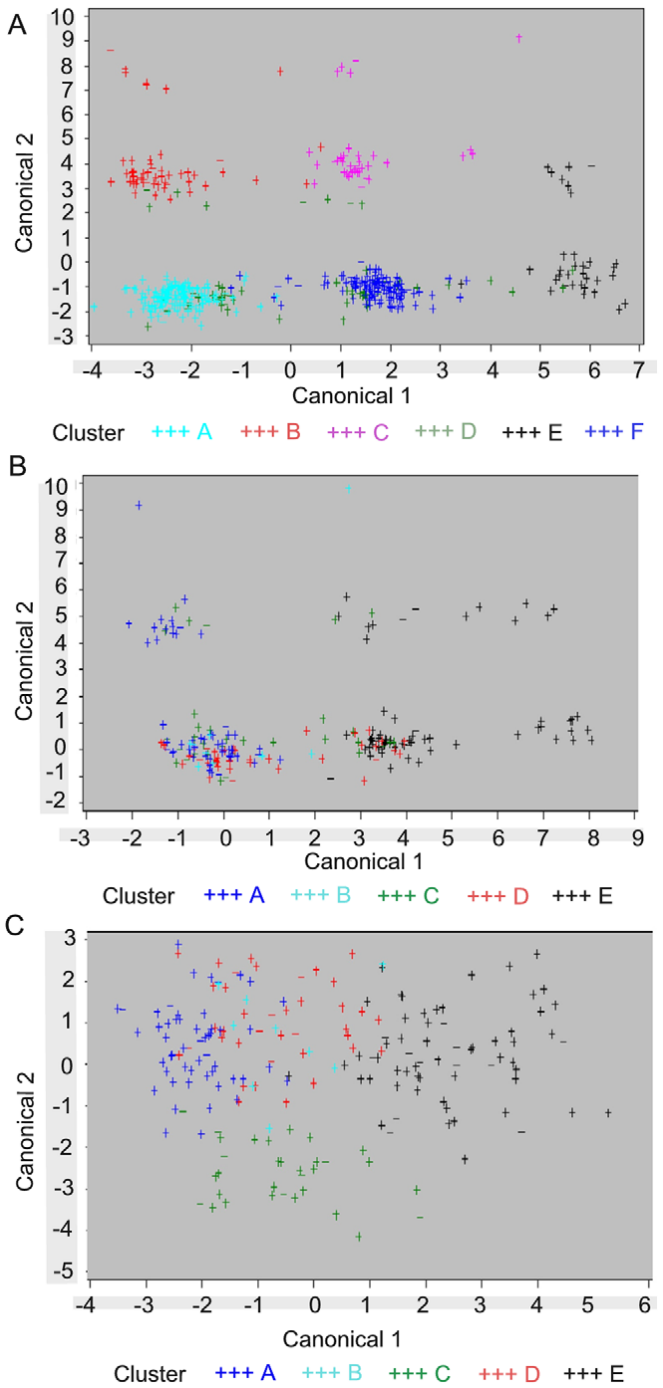
Cluster plots. Cluster plot based on canonical variables for CD patients (**panel A**) and for healthy controls (**panel B**). Cluster plot for healthy controls, when discriminant functions derived from CD patients were applied to the healthy control population (**panel C**).

Canonical discriminant analysis (CDA) was performed in CD patients and healthy controls, separately. The means derived for canonical variable 1 (X1, Y1) and canonical variable 2 (X2,Y2), with (X1, X2) for healthy controls and (Y1, Y2) for CD patients, were: mean value X1: 7.93E-17, SD 2.06; mean value Y1: 7.72E-17, SD 2.61; mean value X2: −2.68E-16, SD 2.36; mean value Y2: 1.96E-16, SD 1.55. When we constructed a plot based on the two main canonical variables for CD patients (Y1, Y2; Figure 2A) and on the two main canonical variables for healthy controls (X1,X2; Figure 2B), a clear difference between both groups in terms of spatial spread of the clusters could be observed (compare Figures 2A and 2B). This supports the existence of differential separation rules for CD patients and healthy controls. Indeed, when canonical variables for healthy controls were computed using the discriminant rule derived for CD patients (analysis 2; see Methods for details), the obtained mean values for the two first canonical variables for CD patients and healthy controls were significantly different (p_manova_<0.0001). Whereas the first two canonical variables nicely separated the CD clusters, the corresponding discriminant function did not clearly separate the clusters in healthy controls (compare Figures 2A and 2C).

Note that one cluster (cluster D in Figure 2A), was less confined to a particular spatial area than the other clusters derived for CD patients. This was to be expected since this cluster was determined by two different branches from the tree plot (see Figure 1B). Healthy controls were also nicely separated on the basis of the two first canonical variables for HC, although to a lesser extent than the CD clusters. This was also reflected in the higher prediction error rate in HC compared to CD patients. Here, cluster B seemed to smear out over the plotted area, which was in agreement with the SNP tree (see Figure 1D), where cluster B does not appear. As stated above, for this cluster, no preference was given to any SNP to determine the cluster.

### Comparing clinical subphenotypes across the identified clusters

The phenotypic characteristics of CD patients in the different genetic-based subgroups are summarized in Table 1. No obvious differences were observed for gender distribution and median age at diagnosis between the different clusters. However, a difference in distribution of disease location (colonic, ileal, or ileocolonic) could be observed between cluster C, and the other clusters (p_Chi^2^_ = 0.02). Prevalence of anal disease, which could occur irrespective of the other disease locations, was lowest in cluster E (15/59 CD patients, 25%), followed by cluster B (33/96, 34%), cluster F (88/239, 37%), cluster A (118/302, 39%), cluster C (26/62, 42%), and cluster D (51/117, 44%). With regards to disease behavior, the prevalence of inflammatory disease behavior was highest in cluster B (57/96 CD patients, 59%), less in cluster E (30/59, 51%) and D (60/117, 51%), followed by cluster F (116/239, 49%) and A (144/302, 48%), and lowest in cluster C (23/62, 37%). This difference (cluster B versus all other clusters) was, although borderline, statistically significant (p_Chi^2^_ = 0.04, OR = 1.59[1.03–2.44]). In addition, cluster B contained the lowest prevalence of non-perianal fistulae (16/96, 17%), compared to 26%–29% in the other clusters (p_Chi^2^_ = 0.03, OR = 0.54[0.31–0.94]). Stenosing disease behavior, and perianal fistulizing disease behavior, tended to be more prevalent in cluster C: 31/62 (50%) CD patients in cluster C had stenosing disease behavior versus 31%–40% in the other clusters (p_Chi^2^_ = 0.04, OR = 1.73 [1.03–2.90]), and 26/62 (42%) CD patients had perianal fistulizing disease behavior versus 29%–31% in other clusters (p_Chi^2^_ = 0.04, OR = 1.71 [1.01–2.90]). Also interesting was the high prevalence of surgery in cluster C: 45 out of 62 CD patients (73%) underwent surgery for their disease, compared to 53%–57% of CD patients in the other clusters. This difference (cluster C versus other clusters) was statistically significant (p_Chi^2^_ = 0.008, OR = 2.13[1.2–3.79]). Multiple logistic regression analysis including all clinical parameters with p<0.1, indicated that cluster B remained independently associated with non-perianal fistulizing disease behavior (p = 0.03, OR = 0.54 [0.31–0.95]; cluster C with perianal fistulizing disease behavior (p = 0.03, OR = 1.82 [1.07–3.14] and need for surgery (p = 0.03, OR = 1.92 [1.07–3.45]; and cluster E with anal disease (p = 0.04, OR = 0.54 [0.29–0.98]. It should be noted however, that all differences seen between the different clusters are modest in magnitude, and would not withstand correction for multiple testing.

On the other hand, random forest (RF) analysis applied to our data gave rise to large inconsistencies between the permutation-based mean decrease in accuracy criterion and the mean decrease Gini impurity criterion, indicating which variable would be the most and least important. From the observation that RF also showed a considerable classification error rate (71,31%) it could be presumed that the given clinical subphenotypes are inadequate or not sufficient to serve as sole class predictors.

## Discussion

Crohn's disease (CD) is a heterogeneous disorder which is classically being classified according to extent and location of disease and its behavior (inflammatory, stenosing or fistulizing) [7], [8]. Except for the association between *NOD2* and ileal disease location, no robust genotype-phenotype associations have been reported for CD. Because of the well-established role for genetics in the etiology of Crohn's disease, we looked whether subgroups of CD patients could also be identified based only on genetic marker information, and treating the clinical subphenotypes as unknown.

The applied technique distinguished six genetic-based subgroups in CD patients. Several genetic-based subgroups were also identified in healthy controls, but these clearly had a different pattern than in CD patients (Figure 2, panels A and B). The discriminant function for separation of clusters in CD patients indeed could not clearly separate the clusters in healthy controls (Figure 2, panel C; and CDA analysis 2). The genetic variants thus clustered in different ways within CD patients and healthy controls. Note that these clusters were derived directly from genetic marker data, completely independent from any *a priori* knowledge about clinical (sub)phenotypes.

Many of the genes/loci found to be associated with Crohn's disease segregate into particular pathways. Two of the key pathways are the autophagy and the Il23/Th17 pathway [12]–[15]. Among the most widely studied and replicated disease loci associated with CD are indeed the autophagy genes *ATG16L1* (rs2241880) [14] and *IRGM* (rs4958847, rs13361189) [15], *NOD2* (rs2066844, rs2066845, and rs2066847) [16], [17], and *IL23R* (rs11209026) [18]. Although these markers were included in this study, except for *IRGM*, they did not clearly pop up in the SNP classification tree as best predicting the identified clusters. In this study, we are actually searching for (a combination of) genetic factors that distinguish CD patients from one another, as opposed to factors that are common to all CD patients (versus healthy controls). These particular SNPs are *strong* susceptibility markers for CD when compared to healthy controls, and could thus be more generally applicable to all CD patients, which could explain why they do not appear in the classification trees. It is indeed believed that genetics of Crohn's disease consists of disease susceptibility genes/loci on the one side, and disease modifying genes/loci on the other. Recent work from the international IBD genetics consortium underscores this idea: re-analysis of GWAS data in function of disease behavior (mild versus aggressive disease) identified a number of SNPs that specifically predispose to a more aggressive disease course in CD. Interestingly, these SNPs were not associated with the disease in the original GWAS (Lee et al., ECCO 2010). Additionally, an important observation in our study was that the SNPs/genes that determine cluster formation in CD do not appear to group in specific pathways: eg *TNFSF15* and *IRGM*, which explain cluster B (Figure 1B) are – to the best of our knowledge – not part of one and the same pathway. It could thus be postulated that there are (more general) disease susceptibility pathways that lead to CD overall as compared to healthy controls: autophagy, Th17 pathway, innate immunity, … . But that the specific disease subphenotype (whether a patient will develop a severe disease phenotype with need for surgery for example) is dependent on single disease modifying genes that are not necessarily playing on different levels of the same pathway. Weersma et al. showed that an increase in the number of risk alleles is associated with an increased risk for Crohn's disease and with a more severe disease course [19]. Still, the absolute difference in the number of risk alleles between patients with Crohn's disease and controls was modest. Also, even in the extensive CD group studied by Weersma et al., the majority of patients carried up until 6 risk alleles, and only 5 CD patients carried 8 risk alleles [19]. It is expected that a plateau phase is reached with respect to the number of risk alleles patients carry. It could therefore be speculated that individuals carrying a specific set of risk alleles clustering in specific pathways (for example in *IRGM*, *ATG16L1* and *NOD2* – all implicated in autophagy), and maybe having been/being exposed to a same environmental factor(s), end up developing CD. When in these patients, a combination of genotypes at different risk loci – all of which part of different pathways – is present (cfr as in the SNP classification tree), the patient will end up developing a specific disease subphenotype, independent from the development of CD overall.

Among the clusters, modest differences in prevalence of disease location and/or behavior could be observed (Table 1). For example, cluster B contained less patients with non-perianal fistulizing disease behavior at last follow-up, and cluster C had a higher prevalence of patients with perianal fistulae and patients with need for surgery. Still, random forest analysis, which estimates the importance of variables in determining classification, showed that CD subgroups found based on genetic data could not be explained adequately by the known clinical (sub)phenotypes. This points to a poor relationship between the genetic-based subgroups and the used clinical subphenotypes. Different explanations could be put forward: (1) A relatively low number of SNPs was included in this exploratory study. It will be important to – in the future – include many more variants in this type of analysis, to look for subgroups within patients. At that time, it will also be important to re-assess the above-mentioned concept of disease susceptibility *pathways* leading to disease *overall*, and *single disease modifying genes* defining the *specific disease course*. (2) As also mentioned above, the genetic variants included in this study are known *susceptibility* SNPs for CD versus healthy controls. The observation that the strongest associated loci to date (*CARD15*, *ATG16L1*, *IL23R*) did not pop up in the classification tree supports the hypothesis that they are indeed more general genes for CD, and might be less usefull to differentiate CD patients. More studies are needed to also discover markers for clinical subphenotypes. (3) CD has a heritability estimate of 50–60% (with λs∼20–35). The proportion of heritability accounted for by the currently known susceptibility loci is about 20% [1]. The currently known variants might explain too little of the genetic part of disease risk to be of much clinical relevance. It is believed that part of the missing heritability is explained by gene-gene interactions, that might be even more important than the independent effects of the single susceptibility genes. A clustering analysis like performed in this study gives a first indication of potentially interacting markers: cfr on one branch of the tree (Figure 1B), one SNP is a potential effect modifier of another SNP on the same branch. Another aspect of the missing heritability are copy number variations, which in the future should be taken up as parameters in this type of analysis. (4) Although speculative, another reason might be that the currently used clinical parameters are not the best way to subclassify CD patients, or at least not in the genetic context. The classification based on extent and location of disease indeed is of limited practical use for clinical application, and for prediction of disease progression. Attempts have been made to better subclassify CD patients using clinical, as well as serological and genetic markers, but diagnostic and prognostic specificity and sensitivity of these methods are generally too low to be useful in clinical practice [7], [20], [21]. Still, from the clinical point of view, the clinical characteristics will continue to be most relevant.

With this study we could identify CD specific genetic-based subgroups, pointing to a non-random clustering of genetic markers in CD patients. The formed CD clusters are likely to contribute to disease pathogenesis. The specific SNP combinations determining the CD clusters could be promising disease (progression) predictors, and deserve future study once internationally ongoing efforts to develop a disability score will be finished. In order to further improve the classification based on genetic markers, in the future, more markers need to be included, even on a genome-wide level. Since this is an exploratory study, validation in independent data sets is necessary. Nevertheless, this technique may serve as a first step to reclassify Crohn's disease. Similar approaches could be interesting also for other complex diseases, and in for example pharmacogenetics where the goal is to find subgroups of patients benefiting most, or being most at risk for side-effects of certain therapies.

## Supporting Information

Methods S1 Supporting methods
(0.06 MB DOC)

Table S1 Summary of included polymorphisms: reference and odds ratio as given in the discovery study.
(0.03 MB XLS)

Table S2 Allele and genotype counts in Crohn's disease (CD) patients and healthy controls.
(0.04 MB XLS)

Table S3 Membership probabilities for Crohn's disease patients.
(0.13 MB XLS)
