# Supplementary material for: Molecular Reclassification of Crohn's Disease by Cluster Analysis of Genetic Variants
Source: PLoS One. 2010 Sep 23;5(9):e12952. doi: 10.1371/journal.pone.0012952 (PMC2944846; doi:10.1371/journal.pone.0012952)
Supplement: Methods S1 — Supporting methods (0.06 MB DOC) [file pone.0012952.s001.doc]

## Methods S1

### Statistical analysis

#### Cluster identification

To identify clusters, latent class analysis (LCA) was applied to the set of 46 genetic markers genotyped in CD patients and healthy controls. LCA assumes that the population is composed of subpopulations (latent classes), each having its distinctive distribution of the included variables (1).

LCA was performed with Multimix, a Fortran program designed to fit latent class models including both continuous and categorical variables (2). Multimix fits a mixture of multivariate distributions using the Expectation-Maximization (EM) algorithm. The use of the EM algorithm also enables the program to handle missing data (3). Hence, for this study every individual - including those with missing genotypes - was included. Class assignment is based on posterior probabilities: in theory, individuals can be allocated to various classes with different probabilities, but final class assignment was made on the basis of highest class (cluster) membership probability.

A limitation of the clustering technique is the determination of the number of latent classes (N) to be included in the model. In many situations there is no *a priori* knowledge, and statistical criteria are required to determine the most optimal value of N (4). In this study, we chose bootstrap p-values to infer N. While penalizing for the number of parameters, information criteria, such as AIC and BIC, only provide a crude rule of thumb to discriminate between groups or statistical models. In particular, models were repeatedly fitted with the number of classes increasing stepwise from 1 to N (model 1, model 2, model 3, …, model N). In order to allow for a global maximum solution, each model was fitted on the original data with different starting values. The run with the highest maximum likelihood was considered as the best run for that model. The optimal value of N was obtained by inspecting the bootstrap-values for the likelihood ratio (LR) test, testing that the population is ‘best’ explained by i+1 classes versus i classes ( i=1, …, N). To limit computation time, only 20 bootstrap samples per LR test were generated. Each sample had the same number of individuals as in the original sample. Bootstrap samples were guaranteed to have the same percentage of missingness as observed in the original data. Each generated sample was submitted to Multimix and the LR results were retained, to construct the bootstrap-based null distribution of the LR test, one for each model pair Model i versus Model i+1 (i=1,…,N)

#### Construction of classification trees

Once subjects were grouped into classes or clusters, on the basis of their available genetic information, we determined the genetic markers that contributed most to the formation of the clusters. To perform this step, i.e. to gain insight into the meaning of the formed clusters, classification trees were generated with the R rpart package (R 2.9.1). Hence, trees were grown using the cluster variable obtained from Multimix as the (categorical) response and SNPs as potential explanatory variates. To avoid over-fitting, we adopted the approach of Breiman et al (5) who suggest to use the “1-SE rule”. This rule relies on using the largest value of the complexity parameter (cp) for which the “cross-validation error” is within one standard deviation of the minimum.

Goodness-of-fit of the obtained classification tree was compared to the cluster assignment of Multimix, by dropping individuals down the classification tree, and by comparing the R tree-based classification of subjects to the Multimix-based one..

#### Testing of the hypothesis of no overall status effect (case vs. control)

To check for the effect of status (case vs. control), canonical discriminant analysis (CDA) was applied to LCA data of both CD patients and healthy controls (SAS 9.1.3). Given two or more groups of observations (here: clusters) with measurements on several variables (here: SNPs), canonical discriminant analysis (CDA) derives a linear combination of the variables that have the highest possible correlation with the groups. The maximal multiple correlation is the first canonical correlation; the second canonical correlation is obtained by finding the linear combination uncorrelated with the first canonical variable that has the highest possible multiple correlation with the groups.

Two analyses were performed as described in Material and Methods.

Note that the Multimix algorithm is adapted to deal with individuals having missing data, still assigning them to a cluster. Canonical coefficients on the other hand are only computed for individuals with complete information.

#### Association of genetic-based clusters and clinical characteristics

To explore whether clinical sub-phenotypes contributed to the cluster formation, Random Forest (RF) analysis was performed in R (R 2.9.1; <http://www.stat.berkeley.edu/~breiman/RandomForests/cc_home.htm>). The patient characteristics and clinical sub-phenotypes used included gender; age at diagnosis; colonic, ileal, ileocolonic, or anal disease location at last follow-up; inflammatory, stricturing, non-perianal fistulizing, or perianal fistulizing disease behavior at last follow-up; and need for surgery. RF analysis estimates the importance of variables in determining classification and can detect possible variable interactions. 1000 trees were build, each time run on 6 classes (6 CD clusters present). For each tree build, 3 variables were taken into account. Two types of criteria were used to check for importance of the clinical contributors: Mean Decrease in Accuracy (permutation-based), and Mean Decrease Gini (impurity) Criterion (6).

## References Methods S1

1. McLachlan, G. and Peel, D. (2000) *Finite Mixture Models*. John Wiley & Sons, New York.

2. Hunt, L. and Jorgensen, M. (1999) Mixture Model clustering using the MULTIMIX program. *Aust N Z J Stat,* **41,** 154-171.

3. Hunt, L. and Jorgensen, M. (2003) Mixture model clustering for mixed data with missing information. *Computational Statistics & Data Analysis,* **41,** 429-440.

4. Spycher, B.D., Silverman, M., Brooke, A.M., Minder, C.E. and Kuehni, C.E. (2008) Distinguishing phenotypes of childhood wheeze and cough using latent class analysis. *Eur Respir J,* **31,** 974-981.

5. Breiman, L., Freidman, JH, Olshen, RA, Stone, CJ (1984) Classification and regression trees. *Wadsworth International Group, Belmont, CA***,** 203-215.

6. Breiman, L. (2001) Statistical modeling: the two cultures. *Statistical science,* **16,** 199-231.
